# Estimate of prevalent diabetes from cardiometabolic index in general Chinese population: a community-based study

**DOI:** 10.1186/s12944-018-0886-2

**Published:** 2018-10-12

**Authors:** Wen-Rui Shi, Hao-Yu Wang, Shuang Chen, Xiao-Fan Guo, Zhao Li, Ying-Xian Sun

**Affiliations:** Department of Cardiology, The First Hospital of China Medical University, 155 Nanjing North Street, Heping District, Shenyang, 110001 China

**Keywords:** Cardiometabolic index, Diabetes mellitus, Dyslipidemia, General population, Obesity

## Abstract

**Background:**

Cardiometabolic index (CMI) defines adiposity based on triglycerides (TG) to high-density lipoprotein cholesterol (HDL-C) ratio and waist-to-height ratio (WHtR). This newly proposed metric has been used to detect multiple cardiovascular risk factors, but data relative to diabetes in the general population are lacking. This study aims to validate CMI’s utility of discriminating diabetes and compares it with other indexes among general Chinese population.

**Methods:**

Analyses were based on a cross-sectional study of 11,478 participants that underwent assessment of metabolic and anthropometric parameters in rural areas of northeastern China in 2013. CMI was calculated by TG/HDL-C × WHtR. Multivariate logistic regressions were performed to clarify CMI’s association with diabetes, ROC analyses were engaged to investigate CMI’s discriminating ability for diabetes.

**Results:**

The prevalence of diabetes was 9.93% in males while 10.76% in females, and increased with CMI’s increment. After full adjustment, each SD increment of CMI had odds ratios (ORs) for diabetes of 1.471 (1.367–1.584) and 1.422 (1.315–1.539) in females and males, respectively. Compared with bottom categories of CMI, the top quartiles had ORs of 3.736 (2.783–5.015) in females and 3.697 (2.757–4.958) in males. The ROC results showed an excellent discriminating power of CMI (AUC: 0.702 for females, 0.664 for males).

**Conclusions:**

An increasing CMI was correlated with higher odds of diabetes, supporting CMI as a useful and economic measure to screen and quantify diabetes in general Chinese population. Monitoring and promoting achievement of dyslipidemia and abdominal obesity based on CMI may improve subclinical and cardiovascular outcomes.

## Background

In recent decades, with the rapid development of economy and society, the prevalence of diabetes mellitus (DM) among Chinese population increased quickly, the overall prevalence of diabetes was 10.9% and that of prediabetes was 35.7% in 2013, more importantly, these data were estimated to increase continuously [1, 2]. Diabetes often involves obesity and dyslipidemia [3–6]. Increased adiposity is the single most important risk factor for diabetes, and Chinese show great tendency to have abdominal obesity, which is more relevant to diabetes [5, 6]. Diabetic dyslipidemia is characterized by high concentration of plasma triglyceride (TG) and small dense low density lipoprotein cholesterol (sdLDL-C) particles together with low high density lipoprotein cholesterol (HDL-C) concentration [3, 4]. These atherogenic lipid profiles contribute partly to the additional risk of developing a cardiovascular disease (CVD) in diabetic patients [7, 8].

Associations between diabetes and obesity indicators or lipid related indexes have been evaluated. A meta-analysis revealed positive associations between diabetes and body mass index (BMI), waist circumference (WC) or waist-to-height ratio (WHtR), and it further identified that indicators of abdominal obesity (including WC and WHtR) performed better than BMI in predicting diabetes [9]. Several studies also revealed that the ratio of total cholesterol to HDL cholesterol (TC/HDL-C) and the ratio of triglyceride to HDL cholesterol (TG/HDL-C) were positively related to the risk of diabetes, especially in young, females and lean people, but the discriminating power of these indexes was still poor [10–12].

Lipid accumulation product (LAP), calculated by following formula: TG (mmol/L) × (WC (cm)-58) for females and TG (mmol/L) × (WC (cm)-65) for males, was proposed to be a continuous marker of lipid over-accumulation [13]. LAP showed great association with cardiovascular events and performed better than BMI in the identification of diabetes [14–17]. But LAP still has some drawbacks. Different formulas are used to calculate LAP, making it inconvenient to compare LAP in patients with different gender. It is also inconvenient that LAP will become minus if the patient’s WC is less than 58 cm in female and 65 cm in male. Moreover, WC cannot represent patient’s status of obesity well because it does not take height into consideration. Thus, WHtR was proposed to replace WC for better representation of abdominal obesity [18–20]. Indeed, WHtR showed a great potential to be a discriminator of coronary heart disease (CHD) risk factors and diabetes [20–23].

Recently, an index called “cardiometabolic index (CMI)” was put forward. As is calculated by TG/HDL-C multiply WHtR, it improves the aforementioned drawbacks of LAP. Early researches showed its close relationship with atherosclerosis in patients with peripheral arterial disease [24]. Later studies also elucidated its association with other diseases including hypertension, left ventricular geometry abnormality, stroke, erectile dysfunction, proofed its value in screening related diseases [25–28]. However, there is no research to evaluate the utility of CMI for discriminating diabetes in general population, especially in Chinese population. A clarification in general Chinese population must be acquired before we put CMI into clinical practice in China. Furthermore, information about comparisons among CMI, LAP, WC and BMI in discriminating diabetes is also rare. Accordingly, this study aims to discover the association between CMI and diabetes, and then explores the usefulness of CMI in discriminating the prevalence of diabetes in a Chinese general population.

## Methods

### Study population

This study originated from a large population-based epidemiological cross-sectional study which was performed between January and August 2013, data of 11,956 permanent residents (age ≥ 35 years) in rural areas of the northeastern China were collected. The full details of the study design and definitions were described elsewhere [27, 29, 30]. We excluded 478 participants because of missing biochemical and clinical covariates. Finally, we recruited 11,478 participants (males: 46.2%) with complete data in the current analysis. The Ethics Committee of China Medical University (Shenyang, China) approved our study protocol. All participants provided written informed consent and all procedures were performed in accordance with the ethical standards. If the participants were disabled, their proxies wrote the informed consents for them.

### Data collection and measurements

Our previous researches fully reported the process about data collection and measurements since they involved in the same survey as this study [27, 29, 30]. Before the beginning of the survey, cardiologists and nurses participated a training and passed a test after that. Information about demographic data, health-related behavior, anthropometric parameters, CVD history, dietary intake, family history of DM and medication usage was collected through a questionnaire by these trained medical staffs.

Data on demographic characteristics, lifestyle risk factors, education level, annual family income and medical history were collected by the questionnaire. Quality control was conducted by a central steering committee with a subcommittee. Race was divided into 2 groups: Han and others. Family history of DM was defined as anyone of the participant’s family members (including father, mother, siblings and children) had diabetes. Medication usage referred to any drugs usage in the past 2 weeks. CVD history included angina pectoris, myocardial infarction, atrial fibrillation, other types of arrhythmia and heart failure. Dietary intake was considered through 3 aspects: vegetable, meat and fatty food. Vegetable and meat consumptions were roughly quantified in terms of kilograms per week while fatty food intake was evaluated by times per week. Vegetable consumption was classified into 4 groups: < 1 kg/w = 0, 1–1.5 kg/w (not including 1.5 kg/w) = 1, 1.5–2 kg/w = 2, ≥2 kg/w = 3. Meat consumption (including red meat, fish and poultry) was also divided into 3 groups: < 0.25 kg/w = 0, 0.25–0.5 kg/w = 1, ≥0.5 kg/w = 2. Lastly, fatty food intake was grouped into 3 parts: rare = 0, 2–3 times/w = 1, ≥4 times/w = 2.

Blood pressure measurements were conducted by 2 randomly selected trained medical staffs after subjects sat and relaxed for at least 5 min. 3 consecutive readings were recorded for every subject and their mean value was used for statistical analyses.

As for the measurements of anthropometric indexes, participants were asked to wear in light clothing without shoes. Calibrated digital scales were used to quantify standard weight to the nearest 0.1 kg. For the measuring of standard height, subjects were asked to hold in a standing position when a calibrated stadiometer was used to quantify readings to the nearest 0.1 cm. Elastic measuring tapes were used to get readings of WC in a horizontal position at a point 1 cm above the umbilicus. All measurements were performed twice and their mean values were taken into analyses.

Processes of storage and methods of laboratory measurements were extensively stated in our prior works [27, 29, 30]. In brief, we used EDTA tubes to collect fasting (12 h overnight) blood samples through venipuncture. Within 1 h after the collection, plasma was separated and frozen at − 20 °C. Then the samples were transported to a certified laboratory for examination. Biochemical analyses were performed by an Olympus AU 640 auto analyzer (Olympus, Kobe, Japan). All laboratory equipment was calibrated and blinded duplicate samples were used.

### Definitions

BMI was calculated as mean weight divided by mean height squared (kg/m^2^). WHtR was defined as WC divided by height in meters. TG/HDL-C was determined as TG divided by HDL-C. CMI was calculated according to the formula: CMI = TG/HDL-C × WHtR. LAP was determined by using the following equation: LAP = TG (mmol/L) × (WC (cm)-58) for females and LAP = TG (mmol/L) × (WC (cm)-65) for males.

Hypertension referred to mean systolic blood pressure (SBP) ≥ 140 mmHg and/or mean diastolic blood pressure (DBP) ≥ 90 mmHg, additionally, participants were also identified as hypertensive patients if they were using antihypertensive medications or had self-reported previous diagnosed hypertension [31]. Diagnosis of diabetes was established if anyone of the following items was reported: fasting plasma glucose (FPG) ≥ 7.0 mmol/L, self-reported previous diagnosis history of diabetes or receiving plasma glucose lowering therapy currently [32].

### Statistical analyses

Analyses were performed separately for each gender. Continuous variables were expressed as mean values±standard deviation (SD) if they showed normal distribution, but expressed as median (interquartile range) if they didn’t possess normal distribution. Categorical variables were described as frequencies (percentages). Comparisons between diabetes group and non-diabetes group were performed with Student’s t tests (normal distribution) or Mann-Whitney tests (not normal distribution) to show differences in continuous variables, and χ^2^ tests were utilized to compare differences of categorical variables between groups. For comparisons of ordinal categorical variables (education level, income, physical activity, vegetable intake, meat intake, fatty food intake) between groups, rank-sum tests were used in order to fully use the ordinal information of these variables. Chi-square linear-by-linear association tests were used to show linear trends of prevalent diabetes across the quartiles of CMI. We used multivariate logistic regression analyses to explore the independent association between CMI (both as a continuous and a category variable) and the occurrence of diabetes. We acquired sex-specific odds ratios (ORs) for 1 SD change of CMI to predict the risk of diabetes. And the results were showed as ORs and 95% confidence intervals (95% CIs). Finally, we employed receiver-operating characteristic (ROC) curves to determine optimal cut-off values of CMI to detect the presence of diabetes in both genders. Discriminative ability of CMI and other variables in detecting diabetes was compared by area under the ROC curves (AUCs). All of the statistical analyses involved were performed by SPSS 25.0 software (IBM corp), Prism 7.0 software (Graphpad software, Inc) and MedCalc version 18.5 (MedCalc software, Belgium), a two-tailed *P* value less than 0.05 indicated statistical significant.

## Results

A total of 11,478 participants (males: 46.2%) were taken into analysis. In this study population, the prevalence of diabetes was given to 9.93% in males and 10.76% in females. Baseline characteristics, clinical and biochemical risk profiles of diabetes were compared between DM group and non-DM group in both genders, the results were showed in Table 1. In both genders, diabetic patients were older than non-DM participants, and diabetic patients showed lower physical activity levels when compared with their counterparts. Furthermore, both in males and females, diabetic patients exhibited significant higher level of SBP, DBP, hypertension rate, weight, WC, BMI, WHtR, FPG, TG, TG/HDL, CMI and LAP than non-diabetic subjects. The percentage of family history of DM, medication usage and CVD history was dramatically higher in diabetic patients than those in non-diabetic participants in both sexes. And for females, diabetic patients also had lower education and income levels, together with a significant disadvantage of height level, as well as lesser intakes of meat and fatty food when compared with their counterparts.

**Table 1:** Characteristics of subjects with diabetes mellitus stratified by sex

Variables	male (*n* = 5308)			female (*n* = 6170)		
	DM (*n* = 527)	non-DM (*n* = 4781)	*p* value^a^	DM (*n* = 664)	non-DM (*n* = 5506)	*p* value^a^
Age (years)	56.43 ± 10.27	54.10 ± 10.81	<.001	58.42 ± 9.11	52.78 ± 10.34	<.001
Race (Han) (%)	497(94.3)	4530(94.8)	0.667	631(95.0)	5221(94.8)	0.820
Education level (%)			0.112			<.001
Primary school or below	241(45.7)	1969(41.2)		457(68.8)	3048(55.4)	
Middle school	224(42.5)	2266(47.4)		169(25.5)	2019(36.7)	
High school or above	62(11.8)	546(11.4)		38(5.7)	439(8.0)	
Income level (CNY) (%)			0.267			0.003
≤ 5000	67(12.7)	642(13.4)		99(14.9)	616(11.2)	
5000–20,000	274(52.0)	2571(53.8)		371(55.9)	3047(55.3)	
> 20,000	186(35.3)	1568(32.8)		194(29.2)	1843(33.5)	
Current smoking (%)	279(52.9)	2743(57.4)	0.051	86(13.0)	936(17.0)	0.008
Current drinking (%)	241(45.7)	2156(45.1)	0.781	12(1.8)	166(3.0)	0.079
Physical activity (%)			<.001			<.001
Low	222(42.1)	1356(28.4)		386(58.1)	2248(40.8)	
Middle	81(15.4)	928(19.4)		130(19.6)	1064(19.3)	
High	224(42.5)	2497(52.2)		148(22.3)	2194(39.8)	
Vegetable intake (%)			0.554			0.516
< 1 kg/w	45(8.5)	448(9.4)		66(9.9)	528(9.6)	
1–1.5 kg/w	120(22.8)	1091(22.8)		165(24.8)	1374(25.0)	
1.5-2 kg/w	146(27.7)	1337(28.0)		202(30.4)	1586(28.8)	
≥ 2 kg/w	216(41.0)	1905(39.8)		231(34.8)	2018(36.7)	
Meat intake (%)			0.202			<.001
< 0.25 kg/w	209(39.7)	1709(35.7)		404(60.8)	2967(53.9)	
0.25–0.5 kg/w	146(27.7)	1468(30.7)		179(27.0)	1476(26.8)	
≥ 0.5 kg/w	172(32.6)	1604(33.5)		81(12.2)	1063(19.3)	
Fatty food intake (%)			0.397			<.001
Rare	398(75.5)	3505(73.3)		611(92.0)	4801(87.2)	
2–3 times/w	97(18.4)	1054(22.0)		51(7.7)	613(11.1)	
≥ 4times/w	32(6.1)	222(4.6)		2(0.3)	92(1.7)	
SBP (mmHg)	154.35 ± 23.60	142.46 ± 22.20	<.001	152.26 ± 24.41	138.65 ± 23.53	<.001
DBP (mmHg)	87.74 ± 12.53	83.33 ± 1.64	<.001	83.72 ± 12.09	80.17 ± 11.35	<.001
Hypertension (%)	392(74.4)	2465(51.6)	<.001	503(75.8)	2496(45.3)	<.001
Height (cm)	166.25 ± 6.47	166.44 ± 6.33	0.522	154.99 ± 6.23	155.68 ± 6.05	0.007
Weight (cm)	72.16 ± 11.66	68.22 ± 11.00	<.001	63.29 ± 10.26	59.90 ± 10.04	<.001
WC (cm)	88.38 ± 9.65	83.29 ± 9.65	<.001	86.11 ± 9.24	80.68 ± 9.64	<.001
BMI (kg/m^2^)	26.04 ± 3.48	24.59 ± 3.53	<.001	26.31 ± 3.83	24.68 ± 3.73	<.001
WHtR	0.53 ± 0.06	0.50 ± 0.06	<.001	0.56 ± 0.06	0.52 ± 0.06	<.001
FPG (mmol/L)	8.20(7.39–10.56)	5.53(5.18–5.92)	<.001	8.22(7.26–10.04)	5.42(5.08–5.81)	<.001
TG (mmol/L)	1.75(1.08–2.80)	1.19(0.84–1.80)	<.001	1.86(1.23–2.83)	1.22(0.87–1.79)	<.001
HDL-C (mmol/L)	1.31 ± 0.39	1.42 ± 0.43	<.001	1.32 ± 0.31	1.42 ± 0.35	<.001
TG/HDL-C	1.43(0.80–2.56)	0.88(0.56–1.50)	<.001	1.45(0.93–2.44)	0.88(0.58–1.42)	<.001
Family history of DM (%)	100(19.0)	317(6.6)	<.001	167(25.2)	461(8.4)	<.001
Medication use (%)	305(57.9)	2215(46.3)	<.001	499(75.2)	3098(56.3)	<.001
CVD history (%)	80(15.2)	483(10.1)	<.001	195(29.4)	970(17.6)	<.001
CMI	0.79(0.40–1.43)	0.43(0.26–0.77)	<.001	0.82(0.50–1.37)	0.45(0.28–0.77)	<.001
LAP (cm·mmol/L)	41.16 (20.28–75.90)	20.46 (10.18–40.32)	<.001	52.73 (30.52–87.81)	26.35 (15.30–46.44)	<.001

Data are expressed as mean ± standard deviation (SD) or median (interquartile range) and numbers (percentage) as appropriate. *DM* diabetes mellitus, *CNY* Chinese currency (1CNY = 0.15 USD), *SBP* systolic blood pressure, *DBP* diastolic blood pressure, *WC* waist circumference, *BMI* body mass index, *WHtR* waist-to-height ratio, *FPG* fasting plasma glucose, *TG* triglyceride, *HDL-C* high density lipoprotein cholesterol, *TG/HDL-C* triglyceride to high density lipoprotein cholesterol ratio, *CMI* cardiometabolic index, *LAP* lipid accumulation product; Medication usage: any self-reported medication used in the past 2 weeks, *CVD history* cardiovascular disease history

^a^Comparisons of category variables between groups were tested by chi-square test and comparisons for continuous variables between groups were tested by Student’s t or Mann-Whitney test

**Fig. 1:**
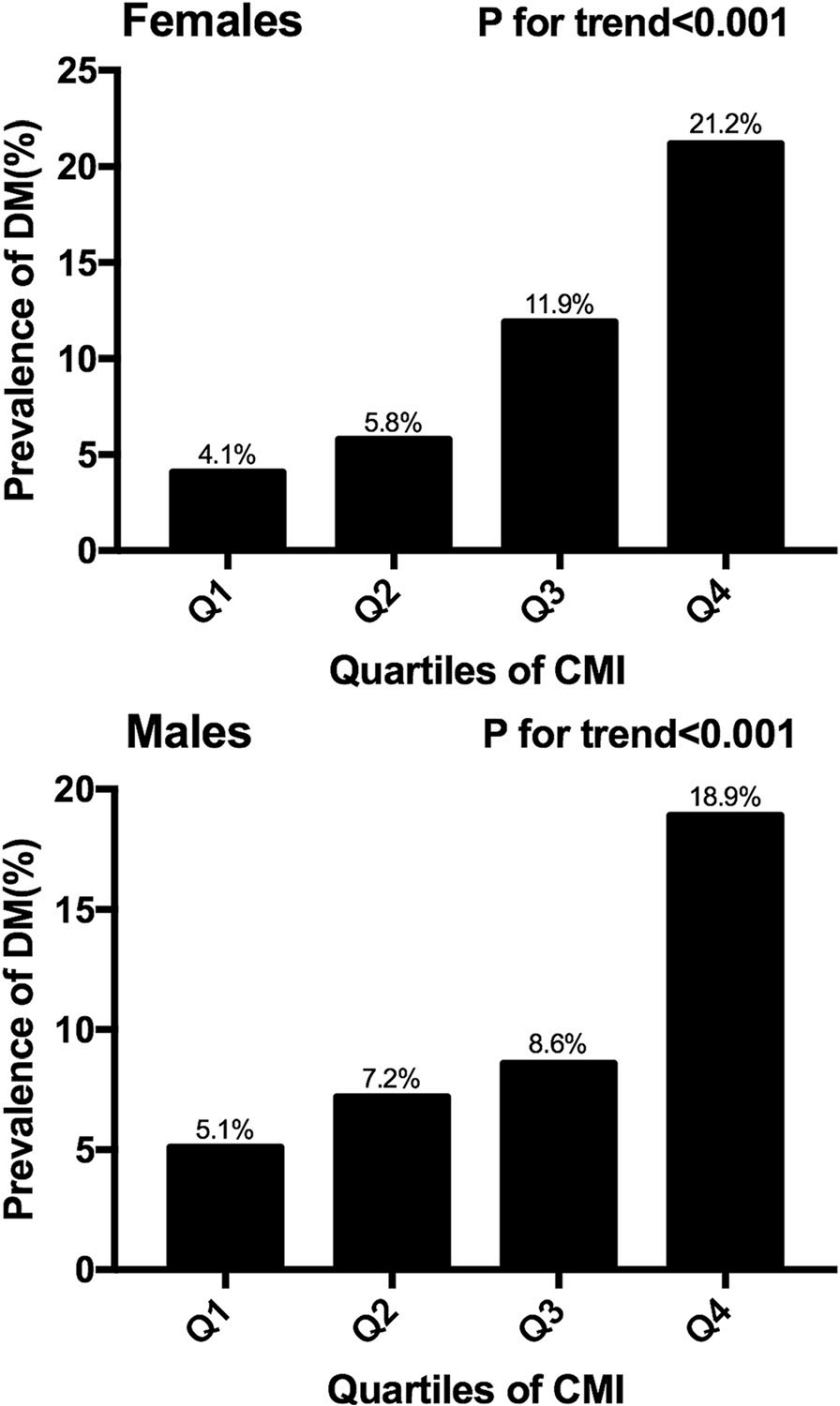
The prevalence of diabetes by quartiles of CMI. Prevalent diabetes increased proportionally across ascending quartiles of CMI in both genders (P for trend< 0.05). Abbreviations: CMI, cardiometabolic index; DM, diabetes mellitus

The prevalence of DM was compared cross quartiles of CMI in each gender as showed in Fig. 1. It exhibited a continuous increment along with the increase of CMI quartiles in both genders (all *p* value for trend< 0.05). When comparing the top with the bottom categories, females showed a 5.2-fold change of diabetic prevalence while males only exhibited a 3.7-fold increment. Results from both genders implicated a strong positive correlation between CMI and diabetic prevalence.

Multivariate logistic regression analyses were carried out to further evaluate the association between CMI and diabetes, and the results were showed in Table 2. CMI had a strong association with diabetes, the odds ratios (ORs) for 1 SD increase of CMI were 1.645 (1.533–1.766) and 1.462 (1.355–1.579) for females and males respectively in crude model. After adjustment of age, race, education levels, income levels and physical activity, the degree of this association changed but still strong, each SD increment of CMI would enhance the risk of DM by 56.8% and 48.7% for females and males respectively. Further adjustment of hypertension, family history of DM, medication usage, CVD history, vegetable intake, meat intake and fatty food intake attenuated the association but not too much, there were still 1.47 and 1.42 times of risk for diabetes in females and males respectively, when CMI had one SD increment. After dividing CMI into quartiles, the risk of prevent diabetes increased robustly with higher CMI quartiles. When comparing top quartiles with bottom categories, risk of prevalent diabetes got 3.74 times increase in females and 3.70 times augmentation in males. *P* values for linear trend were less than 0.05 in both sexes, indicating the linear trends from lowest to the highest quartiles were significant.

**Table 2:** Multivariate logistic regression of CMI for diabetes

variables	Odds Ratio (95%CI)					
	Crude	*P* value	Model 1	*P* value	Model 2	*P* value
Females						
CMI level (per SD change)	1.645(1.533–1.766)	<.001	1.568(1.459–1.686)	<.001	1.471(1.367–1.584)	<.001
Quartiles of CMI						
Q1 (≤0.2962)	1.000(reference)		1.000(reference)		1.000(reference)	
Q2 (0.2962–0.4815)	1.460(1.050–2.031)	0.025	1.323(0.949–1.847)	0.099	1.178(0.839–1.653)	0.344
Q3 (0.4815–0.8263)	3.185(2.370–4.280)	<.001	2.617(1.939–3.532)	<.001	2.145(1.578–2.916)	<.001
Q4 (> 0.8263)	6.331(4.784–8.378)	<.001	4.881(3.667–6.498)	<.001	3.736(2.783–5.015)	<.001
*P* value for trend		<.001		<.001		<.001
Males						
CMI level (per SD change)	1.462(1.355–1.579)	<.001	1.487(1.373–1.611)	<.001	1.422(1.315–1.539)	<.001
Quartiles of CMI						
Q1 (≤0.2728)	1.000(reference)		1.000(reference)		1.000(reference)	
Q2 (0.2728–0.4552)	1.430(1.037–1.971)	0.029	1.420(1.028–1.963)	0.034	1.355(0.976–1.882)	0.070
Q3 (0.4552–0.8285)	1.740(1.276–2.374)	<.001	1.752(1.281–2.398)	<.001	1.576(1.146–2.166)	0.005
Q4 (> 0.8285)	4.305(3.253–5.697)	<.001	4.522(3.394–6.024)	<.001	3.697(2.757–4.958)	<.001
*P* value for trend		<.001		<.001		<.001

Abbreviations: *CMI* cardiometabolic index, *LAP* lipid accumulation product, *OR* odds ratio, *95% CI* 95% confidence interval. Crude: no adjustment; Model 1: adjusted for age, race, education level, income level, current smoking and drinking status, physical activity; Model 2: adjusted for all the factors in Model 1 and hypertension, family history of DM, medication usage, CVD history, vegetable intake, meat intake and fatty food intake

AUCs of ROC analyses showed different ability of CMI, LAP, BMI and WC in discriminating diabetes as summarized in Table 3. In females, LAP had the greatest AUC (0.717, 0.706–0.729) while CMI ranked second (0.702, 0.690–0.713), both indexes showed superior ability in discrimination of diabetes than BMI (AUC:0.626, 95% CI: 0.614–0.638) and WC (AUC: 0.665, 95% CI: 0.653–0.676), implicating a promising value of CMI and LAP for early screening of diabetes. The AUC of LAP was also statistically higher than that of CMI. In males, AUCs of CMI (0.664, 0.651–0.677) and LAP (0.683, 0.670–0.696) were still significantly larger than that of BMI (0.624, 0.611–0.637), LAP also exhibited greater detecting power than WC (AUC:0.650, 95% CI: 0.637–0.663) while CMI did not. AUC of LAP was also significantly higher than that of CMI, though the degree of this superiority was smaller than that in females.

**Table 3:** The area under the ROC curve (AUC) for each index to discriminate diabetes mellitus in females and males

Variables	AUC (95%CI)	*P* value	Cut-off value	Sensitivity (%)	Specificity (%)
Females					
CMI	0.702(0.690–0.713)^a,b^	<.001	> 0.57	68.70	62.20
LAP	0.717(0.706–0.729)^a,b,c^	<.001	> 32.91	73.00	65.50
BMI	0.626(0.614–0.638)	<.001	> 25.57	58.30	61.60
WC	0.665(0.653–0.676)	<.001	> 83.15	64.20	62.50
Males					
CMI	0.664(0.651–0.677)^a^	<.001	> 0.77	51.60	75.00
LAP	0.683(0.670–0.696)^a,b,c^	<.001	> 35.84	57.50	70.93
BMI	0.624(0.611–0.637)	<.001	> 24.56	67.00	52.90
WC	0.650(0.637–0.663)	<.001	> 86.05	60.50	63.10

Abbreviations: *AUC* area under the ROC curve, *95% CI* 95% confidence interval, *CMI* cardiometabolic index, *LAP* lipid accumulation product, *BMI* body mass index, *WC* waist circumference

^a^indicates a significant larger as compared to BMI

^b^indicates a significant larger as compared to WC

^c^indicates a significant larger as compared to CMI

## Discussion

In this large, cross-sectional study of general Chinese population, we reported a positive association between CMI score and prevalence of diabetes after controlling for major confounders. Most importantly, our results also supported CMI to be a premier reproducible screening marker of diabetes without any additional examination or cost. Therefore, our research pointed out an economic way for early controlling of diabetes and primary prevention of subsequent cardiovascular outcomes.

CMI, a recent proposed index that calculated by TG/HDL-C multiply WHtR, originated from LAP (determined by TG and WC) to better reflect the condition of diabetes [33]. CMI also showed its associations with various cardiovascular risk factors besides diabetes, proofed its utility in screening related cardiovascular diseases [25–28]. During the development of diabetic dyslipidemia, serum TG level increases while HDL-C level decreases, leading to a fierce augmentation of TG/HDL-C ratio [34, 35]. Furthermore, TG/HDL-C ratio is considered as a reflection of sd-LDL particles level, which is another important lipid index that changes in diabetic dyslipidemia [36]. Over all, TG/HDL-C is a marker that reflects all 3 characteristics of diabetic dyslipidemia. Another determining factor of CMI is WHtR, which is determined by WC and height. WHtR is considered as a practical and simple indicator of abdominal obesity because it takes height into consideration when evaluating abdominal obesity [37–39]. WHtR has also been identified as a good indicator of diabetes [20, 40, 41]. In conclusion, as a combination of TG/HDL-C and WHtR, CMI is supposed to act as a comprehensive reflection of obesity and dyslipidemia, thus it can be a practical discriminator of diabetes. Results of multivariate logistic regressions showed great similarity with a previous study [33], CMI had strong association with diabetes after adjusting all available confounders. However, our results also showed some differences with the previous study. In the previous study, ORs of females were much higher than that of males, but our results showed similar ORs for both genders. Age difference of the population between the previous study and our study could explain this phenomenon, our female participants had a mean age more than 50 years old, but the mean age of female participants in the previous study was under 40 years old, which means many of our female participants were postmenopausal while most of the female subjects in the previous study were not. Levels of sex hormones (especially estrogen) were identified to associate with increased risk of the prevalent diabetes [42]. Inside the bodies of our female participants, the levels of sex hormones perhaps fell to a degree similar to that of males, so the females could have ORs close to the value of males’ ORs.

As for the results of ROC analyses, our results showed smaller AUC of CMI and LAP for diabetes in both genders than previous studies [17, 33]. This phenomenon might be caused by the difference of race and mean age of the population, another possible reason could be the high rate of hypertensive patients in our participants. Hypertension was elucidated to be another disease closely related to dyslipidemia [43–45], so the discriminating ability of these indexes for diabetes may be influenced. But the exact explanation should be further investigated. Great difference was also discovered in the discriminating ability between CMI, LAP and BMI, WC in both genders, this was different from the confusing results of the previous Japanese study, and confirmed the utility of CMI [33]. Dyslipidemia is a common condition of diabetes, characterized by high TG and sd-LDL-C level with low HDL-C level [34, 35]. CMI and LAP are indexes that take both dyslipidemia and obesity into consideration, so they are reasonable to have greater AUC for discriminating diabetes than indexes that only depict obesity status. Furthermore, TG/HDL-C is an index that reflects diabetic dyslipidemia better than TG alone because it considers all 3 characteristics of diabetic dyslipidemia, WHtR corrects WC with height and is supposed to be a better choice in representing abdominal obesity state. CMI was remodeled from LAP by replacing TG with TG/HDL-C and WC with WHtR respectively. Thus, from the aforementioned information, we can draw that CMI was designed to be an improvement of LAP to discriminate diabetes. But our results showed some differences with our expectation, the AUC of CMI was smaller than that of LAP, and this phenomenon was consistent with a previous study based on Japanese population [33]. The exact reason of this discrepancy between our results and the design of CMI is still unclear, but there is a possible explanation. The hypertension rate of our population was extremely high. It has been proved that hypertension is a risk factor for dyslipidemia (especially HDL-C level) [43–45]. So, the high rate of hypertension probably added additional disturbance on serum HDL-C level in our population, which possibly influenced the discriminating ability of CMI. For this consideration, further studies are needed to evaluate the utility of CMI in a population with lower hypertension rate.

Some mechanical evidences have been discovered to support the results that CMI and LAP did better than BMI and WC in discriminating diabetes. Diabetes is a disease often related to insulin resistance [46]. Insulin resistant fat cells release more free fatty acid (FFA) than normal, and then these excessive FFA fluxes into liver [35]. In the presence of adequate glycogen stores in liver, the influx of excessive FFA will promote overproduction of TG, which in turn increases assembly and secretion of very low density lipoprotein (VLDL), thus the concentration of VLDL-transported TG in serum increases [35, 47]. Increased serum VLDL-C and TG levels further cause decrease of HDL-C level as well as increase of sd-LDL-C level. Under the action of cholesteryl ester transfer protein (CETP), VLDL-transported TG exchanges with HDL-transported cholesteryl ester, and then the levels of cholesterol-rich VLDL and TG-rich HDL in peripheral circulation get increased. TG-rich HDL is further hydrolyzed by hepatic lipase or lipoprotein lipase to form apolipoprotein a-I (ApoA-I), which is filtered out or degraded in kidney [34, 35]. Through a similar mechanism, VLDL-transported TG can be exchanged by CETP with LDL-transported cholesteryl ester to form TG-rich LDL, and then TG-rich LDL will be hydrolyzed by hepatic lipase or lipoprotein lipase to form sd-LDL particles, so the concentration of sd-LDL-C in serum raises [34, 35]. CMI and LAP are 2 indexes that consider abnormal TG change besides indexes of obesity. So theoretically, CMI and LAP are better at discriminating diabetes than BMI and WC, which are only depictions of obesity. Furthermore, CMI takes more characteristics of diabetic dyslipidemia into consideration, so it is supposed to be more effective in discriminating diabetes than LAP. Although our results do not consist with the last point, more studies to compare these 2 indexes are needed.

Our study still has some limitations that need to be mentioned when interpreting our results. First, the cross-sectional design makes our study can only provide an association between CMI and diabetes, causality of this association needs further prospective studies to confirm. Second, our sample was originated from the rural area of northeastern China, the results might not be applicable to populations of other areas or races. Third, the concrete type of diabetes was not identified because our survey did not provide enough information, though the percentage of type I diabetes was expected to be very low in our population. Lastly, considering the epidemiologic feasibility, some other biochemical indexes that are reported to correlate with the presence of diabetes such as plasma insulin, estimated insulin resistance, plasma ghrelin, adiponectin, leptin and cytokines were not collected in our cross-sectional design. As in any observational epidemiologic study, residual confounding by unmeasured risk factors for diabetes is a potential source of bias. Larger studies to examine potential moderators for this association are warranted. Nevertheless, our study still has numerous advantages. First, this was first study to supports use of CMI metric in adulthood as an important predictor of diabetes in the general population of China. And our large sample size allowing for adequate adjustment and subgroup analysis, which showed many differences with the previous Japanese population [33]. Our validation of this association enhanced the applicative value of CMI. Second, we compared CMI and LAP and found out that the discriminating ability of CMI was close but still lower that of LAP, this finding was consistent with the previous study [33]. Thus, whether CMI is a better discriminating index for diabetes than LAP still needs further studies to evaluate.

## Conclusions

In conclusion, our study validated the independent, stable and dose-dependent association between CMI and diabetes in a general Chinese population. Our results enhanced the applicative value of CMI in discriminating diabetes, pointed out an economic way for the prevention of diabetes and subsequent cardiovascular outcomes.

## Abbreviations

ApoA-I: apolipoprotein a-I
AUC: area under curve
BMI: body mass index
CETP: cholesteryl ester transfer protein
CHD: coronary heart disease
CMI: cardiometabolic index
CVD: cardiovascular disease
DBP: diastolic blood pressure
DM: diabetes mellitus
EDTA: ethylenediaminetetraacetic acid
FFA: free fatty acid
FPG: free plasma glucose
HDL-C: high density lipoprotein cholesterol
LAP: lipid accumulation product
ORs: odds ratios
ROC: receiver operating characteristic curve
SBP: systolic blood pressure
SD: standard deviation
sd-LDL-C: small dense low density lipoprotein cholesterol
TC/HDL-C: total cholesterol to high density lipoprotein cholesterol ratio
TG: triglyceride
TG/HDL-C: triglyceride to high density lipoprotein cholesterol ratio
VLDL: very low density lipoprotein
WC: waist circumference
WHtR: waist to height ratio

## References

[1] Shaw J, Sicree R, Zimmet P (2010). Global estimates of the prevalence of diabetes for 2010 and 2030. Diabetes Res Clin Pract.

[2] Wang L, Gao P, Zhang M, Huang Z, Zhang D, Deng Q (2017). Prevalence and ethnic pattern of diabetes and prediabetes in China in 2013. JAMA.

[3] U.K. Prospective Diabetes Study 27. Plasma lipids and lipoproteins at diagnosis of NIDDM by age and sex. Diabetes Care. 1997;20:1683–7. 10.2337/diacare.20.11.1683 9353608

[4] Kannel W (1985). Lipids, diabetes, and coronary heart disease: insights from the Framingham study. Am Heart J.

[5] Yoon K, Lee J, Kim J, Cho J, Choi Y, Ko S (2006). Epidemic obesity and type 2 diabetes in Asia. Lancet.

[6] DeFronzo R, Ferrannini E, Groop L, Henry R, Herman W, Holst J (2015). Type 2 diabetes mellitus. Nat Rev Dis Primers.

[7] Stamler J, Vaccaro O, Neaton J, Wentworth D (1993). Diabetes, other risk factors, and 12-yr cardiovascular mortality for men screened in the multiple risk factor intervention trial. Diabetes Care.

[8] Dunn F (2010). Management of dyslipidemia in people with type 2 diabetes mellitus. Rev Endocr Metab Disord.

[9] Kodama S, Horikawa C, Fujihara K, Heianza Y, Hirasawa R, Yachi Y (2012). Comparisons of the strength of associations with future type 2 diabetes risk among anthropometric obesity indicators, including waist-to-height ratio: a meta-analysis. Am J Epidemiol.

[10] Zhang M, Zhou J, Liu Y, Sun X, Luo X, Han C (2018). Risk of type 2 diabetes mellitus associated with plasma lipid levels: the rural Chinese cohort study. Diabetes Res Clin Pract.

[11] He S, Wang S, Chen X, Jiang L, Peng Y, Li L (2012). Higher ratio of triglyceride to high-density lipoprotein cholesterol may predispose to diabetes mellitus: 15-year prospective study in a general population. Metab Clin Exp.

[12] Wang Y, Koh W, Talaei M, Yuan J, Pan A (2017). Association between the ratio of triglyceride to high-density lipoprotein cholesterol and incident type 2 diabetes in Singapore Chinese men and women. J Diabetes.

[13] Kahn H (2005). The “lipid accumulation product” performs better than the body mass index for recognizing cardiovascular risk: a population-based comparison. BMC Cardiovasc Disord.

[14] Kahn H (2006). The lipid accumulation product is better than BMI for identifying diabetes: a population-based comparison. Diabetes Care.

[15] Hosseinpanah F, Barzin M, Mirbolouk M, Abtahi H, Cheraghi L, Azizi F (2016). Lipid accumulation product and incident cardiovascular events in a normal weight population: Tehran lipid and glucose study. Eur J Prev Cardiol.

[16] Wakabayashi I (2014). Influence of age and gender on lipid accumulation product and its relation to diabetes mellitus in Japanese. Clin Chim Acta.

[17] Wakabayashi I, Daimon T (2014). A strong association between lipid accumulation product and diabetes mellitus in japanese women and men. J Atheroscler Thromb.

[18] Hsieh S, Yoshinaga H, Muto T (2003). Waist-to-height ratio, a simple and practical index for assessing central fat distribution and metabolic risk in Japanese men and women. Int J Obes Relat Metab Disord.

[19] Corrêa M, Thumé E, De Oliveira E, Tomasi E (2016). Performance of the waist-to-height ratio in identifying obesity and predicting non-communicable diseases in the elderly population: a systematic literature review. Arch Gerontol Geriatr.

[20] Zeng Q, He Y, Dong S, Zhao X, Chen Z, Song Z (2014). Optimal cut-off values of BMI, waist circumference and waist:height ratio for defining obesity in Chinese adults. Br J Nutr.

[21] Hsieh S, Yoshinaga H (1995). Abdominal fat distribution and coronary heart disease risk factors in men-waist/height ratio as a simple and useful predictor. Int J Obes Relat Metab Disord.

[22] Hsieh S, Muto T (2006). Metabolic syndrome in Japanese men and women with special reference to the anthropometric criteria for the assessment of obesity: proposal to use the waist-to-height ratio. Prev Med.

[23] Ashwell M, Gunn P, Gibson S (2012). Waist-to-height ratio is a better screening tool than waist circumference and BMI for adult cardiometabolic risk factors: systematic review and meta-analysis. Obes Rev.

[24] Wakabayashi I, Sotoda Y, Hirooka S, Orita H (2015). Association between cardiometabolic index and atherosclerotic progression in patients with peripheral arterial disease. Clin Chim Acta.

[25] Wang H, Chen Y, Sun G, Jia P, Qian H, Sun Y (2018). Validity of cardiometabolic index, lipid accumulation product, and body adiposity index in predicting the risk of hypertension in Chinese population. Postgrad Med.

[26] Wang H, Sun Y, Li Z, Guo X, Chen S, Ye N (2018). Gender-specific contribution of cardiometabolic index and lipid accumulation product to left ventricular geometry change in general population of rural China. BMC Cardiovasc Disord.

[27] Wang H, Chen Y, Guo X, Chang Y, Sun Y (2017). Usefulness of cardiometabolic index for the estimation of ischemic stroke risk among general population in rural China. Postgrad Med.

[28] Dursun M, Besiroglu H, Otunctemur A, Ozbek E (2016). Association between cardiometabolic index and erectile dysfunction: a new index for predicting cardiovascular disease. Kaohsiung J Med Sci.

[29] Li Z, Guo X, Zheng L, Yang H, Sun Y (2015). Grim status of hypertension in rural China: results from Northeast China rural cardiovascular health study 2013. J Am Soc Hypertens.

[30] Wang H, Li Z, Guo X, Chen Y, Chen S, Tian Y (2018). Contribution of non-traditional lipid profiles to reduced glomerular filtration rate in H-type hypertension population of rural China. Ann Med.

[31] Chobanian A, Bakris G, Black H, Cushman W, Green L, Izzo J (2003). The seventh report of the joint National Committee on prevention, detection, evaluation, and treatment of high blood pressure: the JNC 7 report. JAMA.

[32] Classification and Diagnosis of Diabetes. Diabetes Care. 2018;41:S13-S27. 10.2337/dc18-S002 29222373

[33] Wakabayashi I, Daimon T (2015). The “cardiometabolic index” as a new marker determined by adiposity and blood lipids for discrimination of diabetes mellitus. Clin Chim Acta.

[34] Del Pilar SM, Goldberg R (2005). Management of diabetic dyslipidemia. Endocrinol Metab Clin N Am.

[35] Mooradian A (2009). Dyslipidemia in type 2 diabetes mellitus. Nat Clin Pract Endocrinol Metab.

[36] Dobiásová M, Frohlich J (2001). The plasma parameter log (TG/HDL-C) as an atherogenic index: correlation with lipoprotein particle size and esterification rate in apoB-lipoprotein-depleted plasma (FER (HDL)). Clin Biochem.

[37] Dong B, Wang Z, Arnold L, Song Y, Wang H, Ma J (2016). Simplifying the screening of abdominal adiposity in Chinese children with waist-to-height ratio. Am J Hum Biol.

[38] Roswall J, Bergman S, Almqvist-Tangen G, Alm B, Niklasson A, Nierop A (2009). Population-based waist circumference and waist-to-height ratio reference values in preschool children. Acta Paediatr.

[39] McCarthy H, Ashwell M (2006). A study of central fatness using waist-to-height ratios in UK children and adolescents over two decades supports the simple message—‘keep your waist circumference to less than half your height’. Int J Obes.

[40] Jayawardana R, Ranasinghe P, Sheriff M, Matthews D, Katulanda P (2013). Waist to height ratio: a better anthropometric marker of diabetes and cardio-metabolic risks in south Asian adults. Diabetes Res Clin Pract.

[41] Zhao X, Zhu X, Zhang H, Zhao W, Li J, Shu Y (2012). Prevalence of diabetes and predictions of its risks using anthropometric measures in southwest rural areas of China. BMC Public Health.

[42] Muka T, Nano J, Jaspers L, Meun C, Bramer W, Hofman A (2017). Associations of steroid sex hormones and sex hormone-binding globulin with the risk of type 2 diabetes in women: a population-based cohort study and meta-analysis. Diabetes.

[43] Oda E, Kawai R (2011). High-density lipoprotein cholesterol is positively associated with hypertension in apparently healthy Japanese men and women. Br J Biomed Sci.

[44] Kannel W (2000). Fifty years of Framingham study contributions to understanding hypertension. J Hum Hypertens.

[45] Thomas F, Bean K, Guize L, Quentzel S, Argyriadis P, Benetos A (2002). Combined effects of systolic blood pressure and serum cholesterol on cardiovascular mortality in young (<55 years) men and women. Eur Heart J.

[46] Defronzo R (2009). Banting lecture. From the triumvirate to the ominous octet: a new paradigm for the treatment of type 2 diabetes mellitus. Diabetes.

[47] Taskinen M (2003). Diabetic dyslipidaemia: from basic research to clinical practice. Diabetologia.

